# Effects of BTK signalling in pathogenic microorganism infections

**DOI:** 10.1111/jcmm.14548

**Published:** 2019-08-08

**Authors:** Bingjue Ye, Cheng Zhou, Huiting Guo, Min Zheng

**Affiliations:** ^1^ The State Key Laboratory for Diagnosis and Treatment of Infectious Diseases, The First Affiliated Hospital of School of Medicine Zhejiang University Hangzhou China; ^2^ Collaborative Innovation Center for Diagnosis and Treatment of Infectious Diseases Hangzhou China

**Keywords:** bacterium, Bruton's tyrosine kinase, fungus, immunity, parasite, virus

## Abstract

As a cytoplasmic protein tyrosine kinase, Bruton's tyrosine kinase (Btk) is widely considered as a vital kinase in many aspects of different physiologic processes. It is engaged in many important signalling pathways related to the immune response, such as the B cell receptor pathway, pattern‐recognition receptor pathway, and triggering receptor expressed on myeloid cell pathway. Recent studies have increasingly focused on the important role of Btk in various inflammatory diseases, which are related to Btk expression in myeloid innate immune cells, such as macrophages, dendritic cells and neutrophils. Although some investigations have explored the role of Btk in microbial infections, many aspects remain elusive, and some of the results are opposite and controversial. Considering the complicated and multiple roles of Btk in the immune system, we summarized the engagement of Btk signalling in various pathogenic microorganism infections, the possible mechanisms involved and its therapeutic potential in the control of infectious diseases.

## 1 INTRODUCTION

Bruton's tyrosine kinase (Btk) is a cytoplasmic protein tyrosine kinase belonging to the tyrosine kinase expressed in hepatocellular carcinoma (Tec) family of non‐receptor tyrosine kinases (TFKs),^1^ which also include Tec, interleukin (IL)‐2‐inducible T cell kinase (Itk), resting lymphocyte kinase (Rlk) (also called T cell‐expressed kinase), and bone marrow‐expressed kinase (Bmx). In 1952, the phenotype of Btk deficiency was first described by Dr Bruton in a boy who presented with recurrent bacterial infections due to the deficiency in humoural immunity.^2^ This severe primary immunodeficiency is named X‐linked agammaglobulinaemia (XLA). In 1993, the causative gene of XLA, *Btk*, was first identified and isolated.^3^

As the unambiguous causative gene of XLA, many extensive and deep studies on the function of Btk have been performed, focusing on B cells. Recently, many researchers have demonstrated or reviewed that Btk also plays important roles in innate immunity^4^ and is closely related to inflammatory diseases, such as autoimmune and allergy diseases.^5^, ^6^ For example, in rheumatoid arthritis, Btk overexpression occurs,^5^ and inhibition of Btk signalling is an effective approach for its treatment.^7^ Similarly, Btk is required for FcεRI‐mediated activation and histamine release in mast cells and basophils, and the application of Btk inhibitors greatly improves the outcome of allergy diseases.^6^, ^8^ Although there are some studies on the roles of Btk signalling in microbial infections, they are still lacking a comprehensible and in‐depth summary, and the results are uncertain and even conflicting. Therefore, we present the current knowledge on the effects of Btk signalling in pathogenic microorganism infections, including mainly those caused by viruses, bacteria, fungi and parasites. Furthermore, the mechanism and disputes involved and therapeutic implications are also discussed.

## 2 BTK EXPRESSION AND MUTATIONS

In humans, the *Btk* gene is located in the region Xq21.3‐22.1, which contains 19 exons and encodes a 76 kD protein with 659 amino acid residues. The Btk protein comprises five different domains, which are the pleckstrin homology (PH), Tec homology, Src homology (SH) 3, SH2 and kinase domain (SH1) from the N terminus to the C terminus. Among them, SH1 is the catalytic domain.^1^ Btk is generally expressed in all haematopoietic lineages except for T cells and plasma cells, including B cells and all innate immune cells.^9^ Notably, Btk expression in the B cell lineage occurs in a developmental fashion, which shows inconformity during the different stages of B cell development from marrow‐derived haematopoietic stem cells to resting mature cells.^10^ In addition, some evidence indicates that Btk may also be expressed in solid tumours. For example, data based on cDNA sequencing and gene silencing showed that Btk is also expressed in the colorectal adenocarcinoma cell line HT‐29,^11^ and a novel isoform of Btk, Btk‐C, is considerably overexpressed in tumorigenic breast cells rather than in normal breast cells.^12^

In humans, over 800 mutations have been identified to be responsible for the XLA phenotype, including missense, deletion/insertion, nonsense and splice site mutations.^13^ Similarly, the dysfunction of mouse Btk also results in severe X‐linked immunodeficiency (Xid) in mice. The mutation in Xid mice, which is described as R28C and obtained from an arginine to a cysteine in the PH domain, resulted in arrested B cell development.^14^ Poor induction of inflammatory responses also exists in Xid mice because of its significant roles in many myeloid cells.

## 3 ROLE OF BTK IN IMMUNE CELLS AND ITS SIGNALLING PATHWAYS

In XLA patients, B lymphocytes without intact Btk fail to reach the mature state and eventually suffer premature death. Lacking functional circulating B lymphocytes, individuals cannot generate any immunoglobulins in response to antigenic stimulations to develop an effective humoural immune response.^2^, ^15^ Btk dramatically and extensively affects all stages of B cell development, including proliferation, maturation, differentiation, apoptosis and cell migration.^10^ Recent studies have increasingly focused on the awareness of Btk roles in other innate immune cells, such as macrophages, dendritic cells (DCs) and neutrophils.^4^ Btk deficiency decreases the number of monocytes/macrophages. Moreover, defective Btk signalling suppresses FcγR‐mediated cytokine production in monocytes/macrophages but not phagocytosis. In Btk‐deficient mice, DCs are normal in number but defective in antigen presentation and maturation. The population of neutrophils increases significantly in the bone marrow of Xid mice. However, in the absence of Btk, neutrophils are immature, and their functions are impaired. Btk is also required for neutrophil migration, and the expression of the lineage‐determining transcription factors and granule proteins are Btk dependent. Furthermore, Btk is a critical gatekeeper of neutrophil responses because reactive oxygen species production is increased after engagement of Toll‐like receptors (TLRs) or tumour necrosis factor (TNF) receptors in Btk‐deficient neutrophils, which is reversed by the transduction of recombinant Btk.

**Figure 1:**
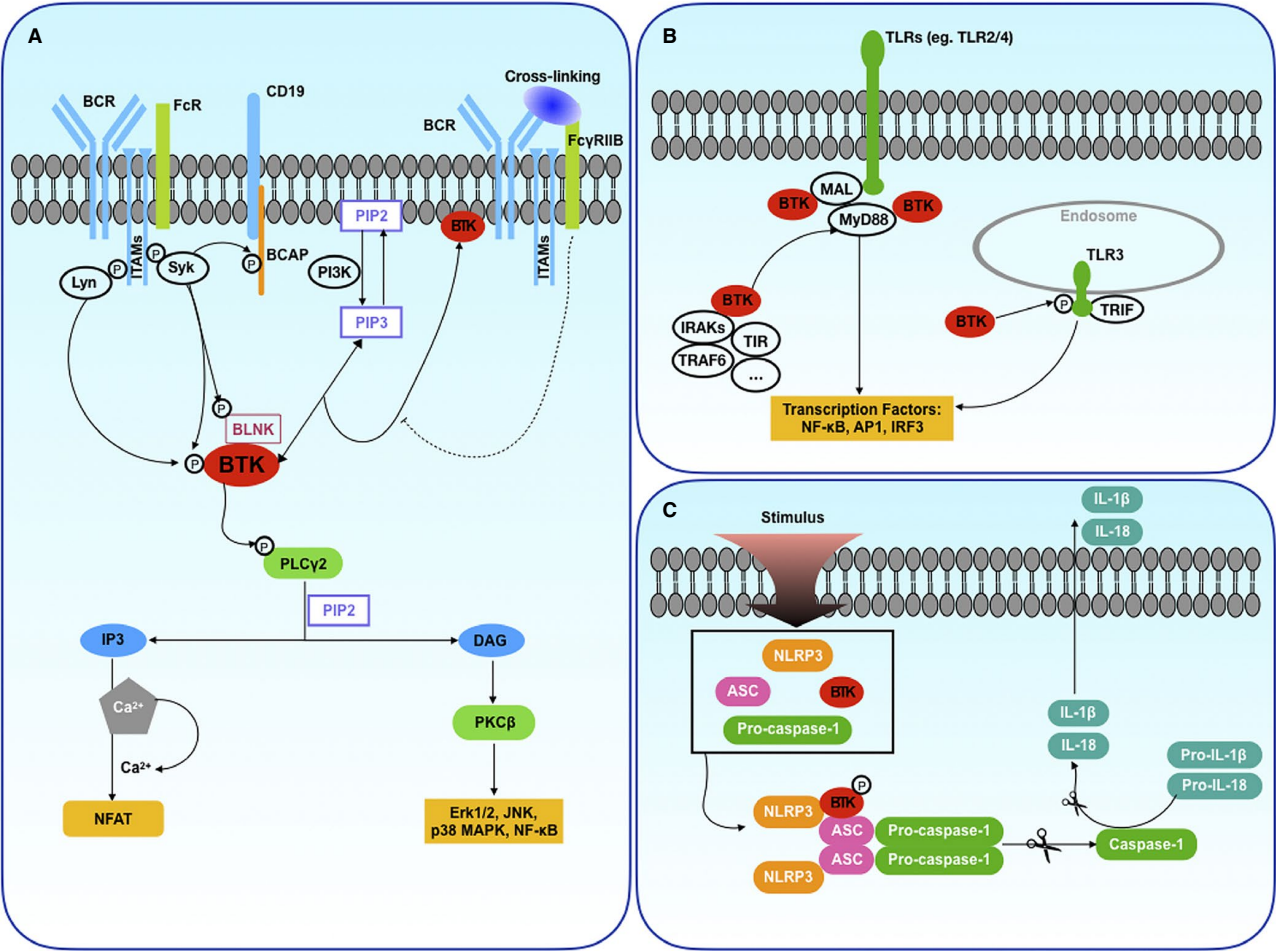
Schematic outline of major Btk signalling cascades. A, In the BCR pathway, upon the activation of BCR, Syk activates PI3K via phosphorylation of BCAP. Then, PI3K phosphorylates PIP2 to generate PIP3, which recruits Btk to the plasma membrane. Btk is phosphorylated by Syk and regulates downstream signalling pathways, such as those of NFAT, Erk1/2, JNK, p38 MAPK and NF‐κB. Similar to the BCR pathway, Btk is also involved in FcR pathways, including activating FcRs and inhibitory FcRs (FcγRIIB). B, In the MyD88‐dependent TLR pathway, after triggering TLRs, Btk cooperates with many proteins, such as MyD88, MAL and IRAKs, and eventually induces transcription factors, including NF‐кB, AP‐1 and IRF3. In addition, Btk can also phosphorylate TLR3 directly. C, In the activation of the inflammasome, Btk hinges NLRP3 with its adaptor ASC to form the functional NLRP3 inflammasome, leading to the activation of caspase‐1, which produces bioactive IL‐1β and IL‐18. See text for details

Lying downstream of BCR, Btk becomes activated by interaction with partner molecules through the PH and SH domains upon activation of BCR with all types of signalling molecules, eventually modifying and maintaining the normal functions of B cells.^16^ Upon BCR activation, immunoreceptor tyrosine‐based activation motifs (ITAMs) in the cytoplasm are phosphorylated by Src‐family protein tyrosine kinases (such as Lyn) and spleen tyrosine kinase (Syk).^17^ At steady‐state, Btk is non‐phosphorylated, and after BCR activation, it is phosphorylated at Tyr551 in the SH1 domain by Syk or Lyn, followed by autophosphorylation at Tyr223 in the SH3 domain.^18^ Meanwhile, Syk facilitates the recruitment and activation of phosphatidylinositol 3‐kinase (PI3K) through phosphorylation of B cell adaptor for PI3K (BCAP), an adaptor protein that interacts with the B cell co‐receptor CD19. PI3K phosphorylates phosphatidylinositol‐4,5‐bisphosphate (PIP2) to generate phosphatidylinositol‐3,4,5‐bisphosphate (PIP3), which recruits Btk to the plasma membrane through linking with the Btk PH domain.^19^ In addition, Syk phosphorylates B cell linker protein (BLNK). In connection with the adapter BLNK, Btk triggers the downstream signalling pathway for calcium release by subsequent phospholipase Cγ2 (PLCγ2) phosphorylation.^20^ Upon the activation of PLCγ2, PIP2 is hydrolysed to inositol triphosphate (IP3) and diacylglycerol (DAG). IP3 activates the transcription of nuclear factor of activated T cells (NFAT) by regulating intracellular calcium levels. DAG mediates the activation of protein kinase Cβ (PKCβ), which eventually induces the activation of many key proteins in cellular physiological processes, such as extracellular signal‐regulated kinases 1 and 2 (Erk1/2), Jun N‐terminal kinase, p38 and nuclear factor кB (NF‐кB) pathway components (Figure 1A). In addition, evidence has shown that Btk is involved in both activating and inhibitory FcR signalling pathways. Similar to the BCR signalling pathway, the Syk‐Btk pathway is also activated following the cross‐linking of activated FcRs. However, cross‐linking of inhibitory FcR (FcγRIIB) and activating receptors such as BCR inhibits the recruitment of Btk, leading to reduced Btk activation^21^ (Figure 1A).

The innate immune system forms the first line of defence to combat foreign or endogenous pathogens, such as foreign microorganisms and molecules released by damaged cells. It is through pattern‐recognition receptors (PRRs) that innate immune cells can sense unusual signals and react rapidly. TLRs are an important family of PRRs that can detect extracellular or intracellular structurally conserved molecular signals derived from pathogens. Several studies have shown that Btk directly participates in activation of key molecules in the TLR pathway. Most TLR signalling pathways except that of TLR3 involve a common and vital protein named myeloid differentiation primary response 88 (MyD88) to maintain recognition function. Upon the activation of those TLRs, MyD88 recruits and transmits the signals to downstream molecules, such as IL‐1 receptor‐associated kinase 1 (IRAK1) and MyD88 adapter‐like (MAL). Btk also cooperates with proteins Toll‐IL‐1 receptor (TIR), MyD88, IRAK1 and MAL to induce activation of a set of transcription factors, including NF‐кB, activator protein‐1 (AP‐1) and interferon regulatory factor 3 (IRF3), suggesting that Btk plays a central role in both innate and adaptive immunities.^22^ However, the TLR3 signalling pathway is MyD88‐independent, in which Btk phosphorylates TLR3 directly and eventually induces the activation of many transcription factors, such as TLR signalling^23^ (Figure 1B). In addition, Btk is also involved in the NOD‐like receptor (NLR), another major family of PRRs, signalling pathway. Being an NLR, NOD‐, LRR‐ and pyrin domain‐containing protein 3 (NLRP3) plays a crucial role in inflammation. Btk is required for IL‐1β release regulated by the NLRP3 inflammasome in macrophages.^24^ In‐depth research shows that Btk hinges NLRP3 with adaptor apoptosis‐associated speck‐like protein containing a CARD (ASC) to form an integral functional complex, leading to the oligomerization of ASC and activation of caspase‐1, which produces bioactive IL‐1β and IL‐18^25^ (Figure 1C).

Bruton’s tyrosine kinase is also involved in the TREM‐1 signalling pathway. Normally, Btk is phosphorylated after TREM‐1 stimulation. However, when the expression of Btk is diminished, Ca^2+^ mobilization and phosphorylation of Erk1/2 and PLCγ1 are reduced upon TREM‐1 triggering. Meanwhile, the production of TREM‐1‐induced pro‐inflammatory cytokines and chemokines is impaired. Therefore, Btk is a positive and essential regulator in TREM‐1 signalling.^26^

## 4 BTK AND PATHOGENIC MICROORGANISM INFECTIONS

### 4.1 Effect of Btk in viral infections

Since Epstein‐Barr virus (EBV) was discovered in African Burkitt lymphoma in 1964, it has been remarkably identified as an oncogenic virus of B cell lymphoma because of its ability to transform resting B cells*.* As EBV immediate‐early genes, BZLF1 and BRLF1 transcription is initiated from a proximal promoter named Zp, whose activation requires intact BCR‐Syk‐Btk pathways.^27^ Moreover, the signalling of latent membrane protein 2A (LMP2A) of EBV, a key protein involved in the regulation of viral latency, is transmitted in both Btk‐dependent and Btk‐independent pathways.^28^ LMP2A^+^ Btk^−/−^ mice exhibit an aggravated Xid phenotype compared with that of Btk^−/−^ littermates, including immature phenotypes and decreased B cell numbers during B cell development, whereas the capability of LMP2A is partially restored in the absence of both Btk and RAG‐1, supporting the production of CD19^+^ IgM^−^ B cells in the bone marrow.^28^ Another study demonstrated that LMP2A enhanced STAT3‐mediated IL‐10 production to promote the survival of EBV‐positive B cell lymphomas through the activation of Btk.^29^ These findings highlight that Btk is a potential drug target for the treatment of EBV‐associated LMP2A‐expressing B cell lymphomas. In addition, as an essential component of EBV, EBV‐encoded nuclear antigen 2 (EBNA2) is also critical for EBV‐infected lymphoblastoid cell growth. A study on the doxorubicin resistance of B cell lymphoma indicated that Btk participates in EBNA2‐induced drug resistance and that the Btk inhibitor ibrutinib can sensitize lymphoma cells to doxorubicin in vitro.^30^ Thus, Btk participates in all the development phases of EBV‐related lymphomas, from infection to tumorigenesis.

In HIV infection, the accessory viral protein Nef links HIV to Btk through the SH3 domain by cell‐based fluorescence detection.^31^ In addition, in HIV‐1‐infected cells, Btk is significantly up‐regulated in its phosphorylated form, and Btk knockdown by small interfering RNA (siRNA) results in the death of infected cells but not uninfected cells. Both specific antibodies and inhibitors, including LFM‐A13 and ibrutinib, can promote the death of HIV‐1‐infected cells and decrease the virus titre markedly.^32^ Similarly, in murine leukaemia virus (MuLV)‐infected Xid mice with defective Btk signalling, the progression of murine acquired immunodeficiency syndrome (MAIDS) is delayed, including abnormal lymphoproliferation and severe immunosuppression.^33^ The studies mentioned above indicated that inhibition of Btk signalling promotes virus clearance and affects symptomatic phenotypes after infection with HIV or MvLV.

In addition, the latest experimental evidence indicates that Btk is involved in influenza A virus (IAV) infection‐associated acute lung injury. Blocking Btk activity reduces weight loss, increases survival and minimizes morphological changes in IAV infection, suggesting that immunomodulatory treatment targeting Btk is an effective approach for controlling influenza‐induced lung injury.^34^

In contrast, selective impairments of DC function are observed in response to oral poliovirus vaccine (OPV) and influenza virus H1N1.^35^ Upon OPV stimulation, monocyte‐derived DCs from XLA patients behave in a dysmature manner and show a significantly decreased production of interferon (IFN)‐α2, IFN‐β and IFN‐λ1, while they are normal in their response to H1N1. Btk is also found to be a critical factor that prevents dissemination of mouse adenovirus type 1 (MAV‐1) in Xid mice because systemically increased viral loads are detected in Xid mice with MAV‐1 infection, which exhibit more serious pathological characteristics, including encephalomyelitis, hepatitis and lymphoid necrosis than the wild‐type (WT) mice.^36^ In vitro, Btk directly phosphorylates TLR3 to regulate the antiviral response, and Btk‐deficient macrophages cultured with dengue virus (DV) show impaired functions in inflammatory cytokine secretion and intracellular DV clearance.^23^ These data indicate that Btk plays an important role in antiviral immune responses.

### 4.2 Effect of Btk in bacterial infections

Given the dysfunction of B cells, XLA patients are susceptible to recurrent bacterial infections and show more severe clinical manifestations.^37^ For example, several case reports have focused on XLA combined with invasive *Klebsiella pneumoniae* polyarticular septic arthritis and *Campylobacter jejuni* systemic infections.^38^, ^39^ Thus, the functions of Btk in a variety of bacterial infections should be determined.

Bruton’s tyrosine kinase plays a protective role against bacterial infections through either innate or adaptive immune responses in vivo or in vitro. Btk‐deficient mononuclear cells from XLA patients demonstrate decreased production of TNF‐α induced by lipopolysaccharide, a component of gram‐negative bacteria.^40^ In Btk‐deficient mice, the anti‐polysaccharide (anti‐PS) response obviously decreases, but the anti‐protein Ig response is normal after immunization with intact *Streptococcus pneumoniae*, indicating that the anti‐PS immune response is Btk dependent.^41^ Compared with WT mice, Xid mice during primary *Coxiella burnetii* infection show severe splenomegaly and higher bacterial burden in the spleens.^42^ In *Borrelia hermsii*‐infected mice, Btk‐deficient mice exhibit weaker T cell‐independent pathogen‐specific IgM responses and higher‐level persistent bacteraemia than WT mice.^43^ In *Staphylococcus aureus*‐infected mice, Btk inhibition negatively regulates IL‐1β‐dependent bacterium clearance through impairing NLRP3 inflammasome activation and blocking IL‐1β release.^25^ In macrophages derived from patients with chronic lymphocytic leukaemia (CLL), the Btk inhibitor ibrutinib impairs the secretion of TNF‐α and affects polarization towards the pro‐inflammatory profile against irradiated *Mycobacterium tuberculosis*. Additionally, ibrutinib‐treated γδ T cells show significantly decreased activation, as indicated by the low expression of the activation marker CD69 and low secretion of IFN‐γ,^44^ providing a better understanding of the risk of infectious complications in ibrutinib‐treated CLL patients.

However, the results of other studies on the role of Btk in bacterial infections are completely different. In response to *Listeria monocytogenes* (Lm), a gram‐positive intracellular bacterium, Btk is activated in bone marrow‐derived macrophages (BMMs), and Btk^−/−^ BMMs show enhanced TNF‐α, IL‐6 and IL‐12p40 secretion, which increases the mean survival time of Btk^−/−^ mice after Lm infection.^45^ In addition, Xid mice infected with virulent *Francisella tularensis* display increased resistance to pulmonary infection, enhanced clearance and significantly greater survival when compared with those of the control mice.^46^ The Btk inhibitor ibrutinib can ameliorate inflammatory myeloid cell responses to protect mice from pneumococcal pneumonia, such as the activation of alveolar macrophages, neutrophil infiltration into the lung and secretion of cytokines.^47^ The above studies suggest that Btk inhibition might be beneficial to the host in bacterial infections. In addition, Musie *et al* revealed that intact Btk signalling is unnecessary in the antibacterial immune response because prevention against *S pneumoniae* by the activation of TLR4 signalling is dependent on T cells but not on intact functional B cells with normal Btk expression.^48^

Several studies have also reported that Btk is closely related to the maintenance of intestinal bacterial balance. Dragoi *et al* reported that Btk expressed in HT‐29 cells can promote *Shigella flexneri* dissemination through phosphorylating the neural Wiskott‐Aldrich syndrome protein in *Shigella* actin tail formation.^49^ Another study demonstrated that fresh stool samples from Btk‐deficient mice display evident alteration of commensal aerobic bacterial homeostasis.^50^

Above all, Btk regulates antibacterial immune responses and intestinal bacterial homeostasis in both innate and adaptive immunities through B cells and other myeloid cells, such as monocytes/macrophages. Thus, Btk performs different functions against various bacterial infections. Almost completely opposite results declare the complexity and diversity of Btk immune functions in bacterial infections. Therefore, Btk is an important regulator between the host and bacteria, but its functions are still elusive.

### 4.3 Effect of Btk in fungal infections

In general, fungal infections are considered opportunistic infections in immunocompromised patients, which are life‐threatening even with optimal medical therapy. In response to fungal spores, endogenous reactive oxygen in macrophages is produced, leading to the rapid phosphorylation of Btk.^51^ Btk‐mediated pathways in macrophages play a vital role in the clearance of fungi through phagocytosis and immunoregulation. When *Candida albicans* is phagocytosed by macrophages, Btk synergized with Vav1 is involved in the formation of phagocytic cups before endocytosis. Simultaneously, macrophages with both Btk and Vav1 deficiency show weakened phagocytosis in this process, indicating the indispensable role of Btk on the formation of the phagosome.^52^ Xid mice are regarded as a vasculitis‐sensitive strain infected with *C albicans*, displaying a high level of inflammatory cytokines, such as IL‐6 and IFN–γ, but a low level of immunosuppressive IL‐10 after activation of various types of pathogen‐associated molecular patterns.^53^ Moreover, Xid mice demonstrated an enhanced susceptibility to *Cryptococcus neoformans* infection, showing increased brain fungal burden, decreased specific serum IgM and impaired alveolar macrophage phagocytosis.^54^ Another study also illustrated that Btk activation in response to *Aspergillus fumigatus* participates in phagocytosis through the TLR9‐Btk‐NFAT pathway.^55^ By contrast, Btk deficiency plays protective roles against intestinal colonization by *C albicans* because decreased infiltrating macrophage numbers and elevated pro‐inflammatory cytokine expression are observed in dextran sodium sulphate (DSS)‐induced Xid mice colitis, suggesting that Btk inhibition combined with *C albicans* colonization can be a possible therapy for the treatment of inflammatory bowel diseases.^56^ Thus, we suppose that during the crosstalk between macrophages and fungi, Btk is phosphorylated rapidly to participate in the formation of phagosomes or regulate the secretion of inflammatory mediators.

### 4.4 Effect of Btk in parasitic infections

Single‐agent treatment with ibrutinib increases the risk of atypical *Pneumocystis jirovecii* pneumonia in CLL patients.^57^ Other published investigations demonstrated that Xid mice are susceptible to infection with *Leishmania amazonensis*,^58^ *Brugia malayi* and *Brugia pahangi*.^59^ These results suggest that the intact function of Btk is required for the spontaneous parasite clearance in vivo and parasite infection control in infected organs.

By contrast, a previous study reported that Xid mice are significantly resistant to infection with *Leishmania chagasi* 60 and *Leishmania major*.^61^ In *Leishmania donovani* infection, the Btk inhibitor promotes host immunity, including an increased number of natural killer T cells producing IL‐4 and IFN‐γ, reduced influx of inflammatory monocytes and enhanced formation of granulomas in the spleen and/or liver, showing excellent availability for the treatment of visceral leishmaniasis.^62^ *Trypanosoma cruzi trans‐*sialidase (TS), a developmentally regulated neuraminidase, is expressed on the cell surface of the parasite, which facilitates adhesion and invasion of *T cruzi*. Btk signalling enhances concanavalin A (ConA)‐induced T cell activation by TS.^63^ Moreover, Btk is required for IL‐17 production from activated B cells stimulated by TS, which is independent of the conventional transcription factors RORγt and Ahr.^64^ Both studies provided new insights into the mechanisms of Btk regulation on *T cruzi* infection, suggesting that inhibition of Btk signalling is a potential approach to treat Chagas’ disease caused by *T cruzi*.

The divergence of Btk function in parasitic infections might be attributed to the different mechanisms of immune responses caused by different parasites and to the different stages of infections.

## 5 DISCUSSION

As summarized above, Btk plays indispensable roles in various pathogenic microorganism infections through both innate and adaptive immunities. However, the roles of Btk in previous studies are different and even opposite. This divergence exists not only in different types of microbial infections but also in infections of the same microbe. We proposed that this diversity is caused by a combination of multifunctional factors.

The host immune system in vivo is a network composed of immune organs, immune cells and immunoreactive substances. The strength of immune responses varies with different host species. Although scientists have tried their best to establish different infectious models in Xid mice, gaps still exist between the immune responses of humans and mice. Xid mice demonstrate considerably milder phenotypic alterations than patients with XLA.^20^ Meanwhile, the number of pathogenic microorganisms is very large, and every pathogenic species has its special infectious processes and induces different immune responses, which might explain the completely different roles of Btk in some studies. For example, as mentioned above in parasitic infections, different *Leishmania* subspecies determine different infection outcome in mice.^58^, ^59^, ^60^

Aiming at Btk functions, most researchers have used three basic tools, including Xid mice, Btk inhibitors and Btk siRNA. Although Btk can be successfully inhibited by either a Btk inhibitor or Btk siRNA, the differences between pharmacological inhibition and genetic Btk deficiency cannot be overlooked. Conclusions about its roles in different infections must be drawn more carefully because abnormal functions of B cells and other innate immune cells already exist in the Btk‐deficient host, whereas pharmacological inhibition focuses on blockade of the phosphorylation site and affects its kinase activity in a normal immune system. Since there are few investigators using the Xid mouse and Btk inhibitor simultaneously to explore the effects of Btk in specific microbial infections, it is difficult for us to obtain affirmative conclusions about the roles of Btk in microbial infections from different studies. This phenomenon also exists in other studies. For example, receptor interacting protein kinase 1 has also been investigated by scientists using knockout mice and a specific kinase inhibitor to obtain contrary outcome in ConA‐induced hepatitis.^65^

Every domain of Btk has a site for specific molecules to participate in various signalling pathways related to different physiological activities, which makes Btk function complex and variable. Meanwhile, Btk, Tec and Itk partially overlap not only in expression patterns but also in functions,^66^ and Tec has been reported to partially compensate for Btk functions in mice.^10^ Although the effects of Btk signalling on pathogenic microorganism infections are not exactly consented upon, there is no doubt about its importance in maintaining the balance of the immune microenvironment. Many studies have shown that enhanced Btk function plays an important role in inflammatory diseases, such as IAV‐induced acute pneumonia.^34^ Therefore, we can control inflammatory processes by regulating Btk activity during different periods of disease to alleviate tissue damage and avoid organ failure. Fortunately, the Btk inhibitor uniformly shows impressive efficacy in controlling the inflammatory processes of several microorganism infections in vivo and/or in vitro (shown in Table 1).

**Table 1:** Therapeutic applications of Btk in pathogenic microorganism‐related diseases

Subjects	Disease model	Btk inhibitor	Therapeutic applications
EBV	LMP2A‐positive B cell lymphoma lines	Ibrutinib	Btk was a new pharmaceutical target to treat EBV‐associated lymphomas that express LMP2A.
	Doxorubicin resistance of B cell lymphoma	Ibrutinib	The Btk inhibitor sensitized EBNA2‐positive DLBCL cells to doxorubicin.
HIV	HIV‐1‐infected cells	LFM‐A13, Ibrutinib	Btk was up‐regulated in HIV‐1‐infected cells, and antibody treatment, inhibitors and Btk knockdown by siRNA showed anti‐HIV effects.
IAV	IAV‐infected mice	Ibrutinib	The Btk inhibitor has a protective effect in IAV‐induced acute pneumonia.
*Staphylococcus aureus*	*Staphylococcus aureus* infection in vivo and in vitro	Ibrutinib	Βtk could be a potential drug‐target for the treatment of NLRP3 inflammasome‐linked inflammation.
*Streptococcus pneumoniae*	*Streptococcus pneumoniae‐*infected mice	Ibrutinib	Ibrutinib has the potential to protect against pneumonia.
*Leishmania donovani*	*Leishmania donovani*‐infected mice	Ibrutinib	Ibrutinib could be a new effective drug for visceral leishmaniasis infection.

Although inhibition of Btk activity is indeed beneficial in the treatment of those microorganism infections in the laboratory, evidence from clinical practice remains lacking. Due to the intricacies of Btk signalling in pathogenic microorganism infections, there is still much work to be done to identify its definite function in the antimicrobial response for better understanding and estimation of the clinical application of Btk inhibitors.

## 6 CONCLUSION

The biological characteristics of Btk in B cells have been well defined since its discovery in the 1990s. In recent years, the roles of Btk in other cell types have attracted increasing attention. Provided with Xid mouse and Btk‐specific inhibitors, numerous studies in vivo and in vitro have revealed that Btk plays key roles in many important pathophysiological processes, suggesting that Btk is closely related to a broad range of diseases. In this review, we summarize multiple effects of Btk in the response to clear pathogenic microorganisms and show that Btk might be a new effective drug target for the therapy of some infectious diseases. Because Btk has many uncertain or opposite characteristics in pathogenic microorganism infections, future studies should focus on elucidating the definite role of Btk in infectious diseases and the involved mechanisms to develop effective treatment using Btk‐specific inhibitors in clinical practice. To this end, accurate and advanced techniques should be applied to elucidate the effects of Btk in various microbial infections in the near future, such as single‐cell sequencing that can reveal the altered phenotypes and functions of different immune cell subgroups from all angles in detail.

## CONFLICT OF INTEREST

The authors confirm that there are no conflicts of interest.

## AUTHOR CONTRIBUTIONS

Both Bingjue Ye and Cheng Zhou drafted the manuscript, Huiting Guo supplemented the manuscript. Min Zheng reviewed the manuscript and provided revisions. All authors reviewed the manuscript.
